# Prognostic significance of ING3 expression in patients with cancer: A systematic review and meta-analysis

**DOI:** 10.3389/fonc.2023.1090860

**Published:** 2023-02-09

**Authors:** Zehan Li, Shengchao Xu, Lin Chen, Shuqi Huang, Xieyida Kuerban, Tianyu Li

**Affiliations:** ^1^ The Department of Surgery, the First Dongguan Affiliated Hospital of Guangdong Medical University, Guangdong, China; ^2^ The Department of Surgery, Guangzhou Medical University, Guangdong, China

**Keywords:** cancer, gene, biomarker, prognosis, meta-analysis

## Abstract

**Background:**

It has been reported that ING3 inhibits the progression of various cancers. However, some studies have shown that it promotes the development of prostate cancer. The purpose of this study was to investigate whether ING3 expression is associated with the prognosis of patients with cancer.

**Materials and methods:**

PubMed, Cochrane Database, Embase, Medline, ScienceDirect, Scopus and Web of Science were searched until September 2022. The hazard ratio (HR)/odds ratio (OR) and 95% confidence interval (95% CI) were calculated using Stata 17 software. We used the Newcastle-Ottawa Scale (NOS) to assess the risk of bias.

**Result:**

Seven studies involving 2371 patients with five types of cancer were included. The results showed that high expression of ING3 was negatively associated with a more advanced TNM stage (III-IV vs. I-II) (OR=0.61, 95% CI: 0.43-0.86), lymph node metastasis (OR=0.67, 95% CI: 0.49-0.90) and disease-free survival (HR=0.63, 95% CI: 0.37-0.88). However, ING3 expression was not associated with overall survival (HR=0.77, 95% CI: 0.41-1.12), tumor size (OR=0.67, 95% CI: 0.33-1.37), tumor differentiation (OR=0.86, 95% CI: 0.36-2.09) and gender (OR=1.14, 95% CI: 0.78-1.66).

**Conclusion:**

This study showed that the expression of ING3 was associated with better prognosis, suggesting that ING3 may be a potential biomarker for cancer prognosis.

**Systematic review registration:**

https://www.crd.york.ac.uk/prospero/, identifier (CRD42022306354).

## Introduction

The ING family, including ING1, ING2, ING3, ING4 and ING5, is a group of type II tumor suppressors. The ING family is involved in DNA repair, chromatin remodeling, cell cycle control, senescence and apoptosis through multiple mechanisms (1–4). The highly conserved plant homeodomain (PHD) is considered to be the main domain of ING protein (5, 6). PHD domains play a key role in the function of epigenetic regulatory proteins, which are known as chromatin remodeling factors and perform a function in gene expression (7). The epigenetic regulatory protein is added at lysine 4 (H3K4me3) after binding to histone H3 with 3 methyl groups through the PHD domains. H3K4me3 is a biomarker of active transcription, in this way ING proteins participate in cell growth regulation (8, 9).

ING3 encodes a 418 amino acid protein located on chromosome 7q31 with a nuclear localization sequence and a conserved carboxy-terminal PHD, the latter of which is thought to play an important role in inhibiting tumorigenic processes (10–13). ING3 has long been considered as a tumor suppressor gene because of its biological functions in limiting cell growth, controlling cell cycle arrest, and initiating apoptosis in a P53-dependent pathway (14). However, Nabbi et al. found that ING3 promotes cell proliferation and functions as an oncogene in prostate cancer (15). In addition, according to another study, ING3 is necessary for the growth of breast and ovarian cancer cells (16). Unlike ING1 and ING2, which can act as AR corepressors to inhibit androgen signaling, ING3 can act as an AR coactivator and thus play a role in prostate cancer (17, 18).

Due to the contradictory findings above, the predictive function of ING3 remains unknown in most investigations. Therefore, the purpose of this study was to determine whether ING3 expression is associated with cancer prognosis. In this study, we performed a systematic review and meta-analysis to summarize the results of global studies predicting the clinical outcomes of ING3 expression in cancer patients.

## Materials and methods

This study was designed and implemented according to the Preferred Reporting Items for Systematic Reviews and Meta-Analyses (PRISMA) criteria (19). This study aimed to determine whether ING3 expression is associated with cancer prognosis. The study was registered in the International Systematic Review Prospective Register. (PROSPERO 2022 CRD42022306354).

### Selection criteria

Inclusion and exclusion criteria were based on Population, Intervention, Comparison, Outcome (PICO) criteria.

The following criteria were used to determine the eligibility for candidate studies: (1) All included patients were diagnosed with cancer by established means. (2) A well-established molecular approach, such as PCR, was used to determine the expression level of ING3 in the included patients. (3) The included literature provided data that could be used for prognostic analysis such as HR and 95% CI for overall survival (OS)/disease-free survival (DFS), or data that could be used to estimate HR and 95% CI, such as Kaplan-Meier survival curves. (4) The study provided an association between ING3 expression and clinicopathological characteristics, such as gender, lymph node metastasis, TNM stage, tumor size, and tumor differentiation.

Articles that met the following criteria were excluded: (1) Clinicopathological characteristics (such as gender, lymph node metastasis, TNM stage, etc.) were not provided in the article. (2) Non-clinical experiments, such as *in vivo* or *in vitro* experiments. (3) Articles were review articles, case reports, letters, clinical guidelines, etc. (4) The article did not provide or could not extract HR and 95% CI. (5) For studies with multiple publications, the study that provided the most data was selected.

All articles that met the inclusion criteria and did not meet the exclusion criteria were read in full. Articles that had been read in full text and met the criteria underwent data extraction. All publications were independently screened by two authors (Li, Xu), and any disagreements were resolved after discussion between the two authors or by consulting a third reviewer (Chen).

### Literature search

We conducted a literature search on the relationship between ING3 expression and patient prognosis up to September 2022. The following databases were searched: PubMed, Cochrane database, Embase, Medline, ScienceDirect, Scopus, and Web of Science. The ClinicalTrials was also searched for any related trials. No filters were used in the search process. We also searched Google Scholar to see if there was any grey literature.

The following keywords were used for the search: "tumour*", " tumor*", " malignan*", " carcinom*", "neoplas*", "cancer*", "ING 3", "ING3", "inhibitor of growth 3", "inhibitor of growth protein 3", "ING protein" 3".

Taking PubMed as an example, the specific search strategies were as follows:

#1 ING3[Title/Abstract] OR ING 3[Title/Abstract] OR inhibitor of growth 3[Title/Abstract] OR Inhibitor of growth protein 3[Title/Abstract] OR ING protein 3[Title/Abstract]
#2 "Neoplasms"[Mesh]
#3 tumour*[Title/Abstract] OR tumor*[Title/Abstract] OR malignan*[Title/Abstract] OR carcinom*[Title/Abstract] OR neoplas*[Title/Abstract] OR cancer*[Title/Abstract]
#4 #2 OR #3
#5 #1 And #4

We used Endnote X9 software to exclude duplicate documents. We first excluded articles with the same author, year, title, journal, volume, issue and page, and then gradually narrowed the criteria until only articles with the same title were excluded. Each time we double-checked the excluded articles, we believed that by doing so we could avoid excluding any articles relevant to this study.

### Data extraction

The following data were extracted from the study: author, year, tumor type, sample size, country, follow-up time, lymph node metastasis, TNM stage, tumor size, tumor differentiation, the cut-off value of hazard ratios (HRs) and 95% confidence intervals (CIs) of ING3 for overall survival (OS) and disease-free survival (DFS). All data were extracted separately by two authors (Li, Xu).

### Quality assessment

The Newcastle-Ottawa Scale (NOS) was used by two authors (Li, Xu) to assess the quality of each study (20), and any disagreements were resolved through mutual discussion or consultation with a third reviewer (Chen). Object selection, comparability and outcome were the most important evaluation items. Quality evaluation scores ranged from 0 (lowest) to 9 (highest); studies with NOS scores greater than 6 were regarded as high quality.

### Statistical analysis

HR is the best performance indicator for measuring time-to-event outcomes (21). The ING3 expression was treated as a binary variable for the sake of this study (high expression vs low expression). To obtain an overall estimate for the DFS and OS analyses, the HRs and 95% CIs were calculated for each study. HRs and 95% CIs were extracted directly from the included studies or transformed from Kaplan-Meier survival curves using the digitizing program Engauge Digitizer (22). We used two endpoints in this study. The primary endpoint was the relationship between ING3 expression and overall survival (OS) and disease-free survival (DFS). The secondary endpoint was the relationship between ING3 expression and clinicopathological features, such as gender, lymph node metastasis, TNM stage, tumor size, and tumor differentiation. Due to conflicting findings in prostate cancer, to explore whether ING3 plays a different role in prostate cancer, we decided to perform a subgroup analysis by cancer type (prostate cancer vs. other) before the study started. To detect statistical heterogeneity between various studies, the Higgin's I^2^ statistics and the Cochran's Q test were used (23). Heterogeneity was considered statistically significant at I^2^>50%. When I^2^>50%, a random-effects model was used to evaluate the pooled results; otherwise, a fixed-effects model was used to analyze the pooled results. The combined HRs and 95% CIs from each included study were used to assess the effect of ING3 expression on prognosis. In general, a combined HR > 1 is considered to indicate a significant association with poor prognosis, and a combined HR < 1 indicates a better prognosis. A sensitivity analysis was performed to assess the stability of the combined data and to pinpoint the cause of any heterogeneity. If the results are consistent, they are considered robust; otherwise, they will be regarded with caution. Begg's and Egger's linear regression tests were used to investigate publication bias. All the above analyses were performed using STATA 17.0 and Review Manager 5.4.

## Result

### Study selection

**Figure 1:**
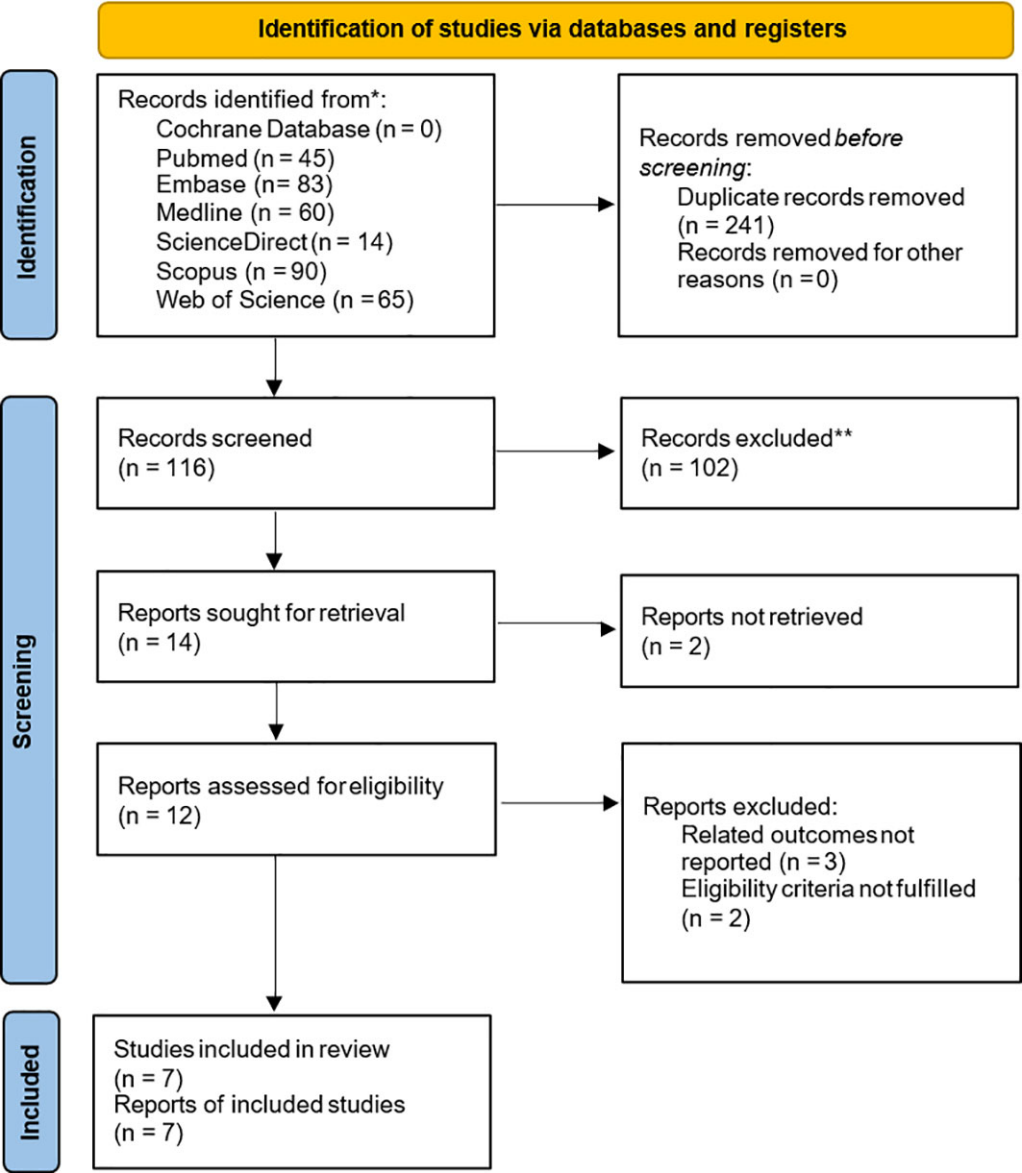
Flowchart of literature search and study selection. Flowchart of literature search and study selection. *Consider, if feasible to do so, reporting the number of records identified from each database or register searched (rather than the total number across all databases/registers). **If automation tools were used, indicate how many records were excluded by a human and how many were excluded by automation tools.

As shown in the literature search flow chart (**Figure 1**), up to September 2022, there were 357 literature from PubMed, Cochrane Database, Embase, Medline, ScienceDirect, Scopus and Web of Science. No relevant studies were found on the trial registration website (clinicaltrials.gov) or Google Scholar. Among these studies, 241 were identified as duplicates and subsequently excluded. 102 publications were excluded after reading the titles and abstracts. Five publications were excluded after reading the full text, including three that did not provide relevant outcomes (12, 24, 25) and two that did not fully meet the inclusion criteria (26, 27). Finally, seven publications were included in the study based on the inclusion and exclusion criteria (14, 15, 28–32).

### Characteristics of the included studies and quality evaluation

Among the eligible studies, two were related to prostate cancer (15, 30), two were related to breast cancer (28, 29), and the remaining three were related to hepatocellular carcinoma (31), head and neck cancer (32), and colorectal cancer (14). The majority of studies (n = 4) were conducted in China, followed by Canada (n = 2) and Japan (n = 1). Three studies provided HRs and 95% CIs in the article, and for the remaining studies, we derived HRs and 95% CIs from Kaplan-Meier curves. The systematic review and meta-analysis comprised 2317 patients from seven selected publications, with sample sizes ranging from 71 to 1081. **Table 1** summarizes the characteristics of the seven published studies. All patients with malignancy were diagnosed based on histology. Tissue samples were obtained from tumors and adjacent normal tissues, and ING3 expression was evaluated by qRT-PCR.

**Table 1:** Study characteristics of the studies included in the systematic review and meta-analysis.

First Author	Year	Cancer	Sample size	Region	Stage	Survival analysis	ING3(H/L)	Therapy	Follow up	Survival endpoints	OS HR	DFS HR
Amal Almami	2016	prostate cancer	304	Canada		Univariate	214/90		100m	OS	0.67	
Arash Nabbi	2017	prostate cancer	256	Canada		Multivariate			100m	OS	2.42	
Hai-Yan Yang	2012	hepatocellular carcinoma	120	China	I-IV	Univariate	50/70		12-124m	OS/DFS	0.24	0.84
Hui-meng LI	2021	breast cancer	1081	China		Univariate	271/810		250m	OS	0.87	
Mehmet Gunduz	2008	head and neck cancer	71	Japan	I-IV	Univariate	34/37	Radiation therapy/Chemotherapy	2-129m	OS/DFS	0.97	0.78
Wen-Feng Gou	2014	colorectal carcinogenesis	274	China	I-IV	Univariate	163/111		0.9-145m	OS	1.22	
Xiao-yan Wu	2021	breast cancer	211	China	I-III	Multivariate	98/113		60m	OS/DFS	0.56	0.56

All included articles were assessed using the NOS scale, with scores ranging from 7 to 9. Three publications had a NOS score of 7, two publications had a NOS score of 8, and two publications had a NOS score of 9, for a total of seven publications with a median score of 8 (**Table 2**).

**Table 2:** NOS score of the included studies.

First Author	Year	Selection	Comparability	Outcome	Total score
Amal Almami	2016	4	1	2	7
Arash Nabbi	2017	4	1	2	7
Hai-Yan Yang	2012	4	2	2	8
Huimeng LI	2021	4	1	2	7
Mehmet Gunduz	2007	4	2	3	9
Wen-feng Gou	2014	4	2	3	9
Xiaoyan Wu	2021	4	2	2	8

### Primary endpoint: Relationship of ING3 to DFS and OS

**Figure 2:**
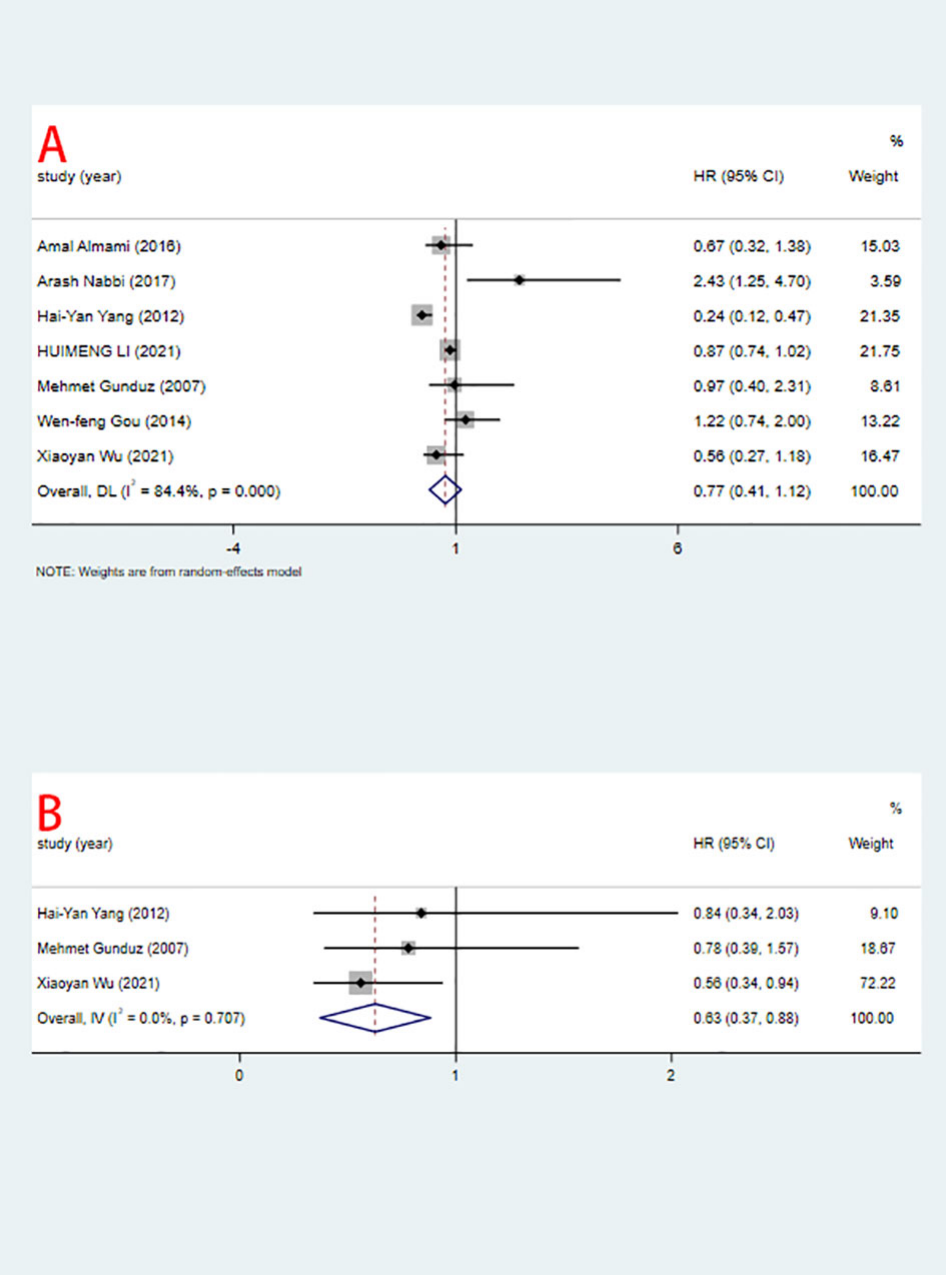
Relationship between ING3 expression and OS **(A)**, DFS **(B)** (high vs low).

Seven studies including 2371 patients reported HRs for OS based on ING3 expression levels. As shown in **Figure 2A** and **Table 3**, given the significant degree of heterogeneity (I^2^=84.4%), a random-effects model was used. In most studies, ING3 expression seemed to be associated with a better prognosis; however, since their 95% CI crossed the null effect line (HR=1), these results lacked statistical significance. In one of these studies, high expression of ING3 indicated poorer overall survival, while in another study, high expression of ING3 indicated a better prognosis. In the remaining studies, no relationship was found between ING3 expression and patient prognosis, as the range of 95% CI crossed 1. The combined data showed that ING3 expression was not associated with prognosis in these patients, and the pooled HR for OS was 0.77 (95% CI: 0.41–1.12, *P* = 0).

**Table 3:** Relationship between ING3 expression and clinicopathological data.

Clinicopathological characteristics	Studies	OR/HR and 95% CI	Effects model	Heterogeneity (p; I^2^)	P value
Overall survival	7	0.77 (0.41-1.12)	Random	0; 84.4%	0
Disease-free survival	3	0.63 (0.37-0.88)	Fixed	0.707; 0%	0
TNM stage (III-IV VS I-II)	4	0.61 (0.43-0.86)	Fixed	0.87; 0%	0.005
Lymph node metastasis (Yes VS No)	4	0.67 (0.49-0.90)	Fixed	0.33; 13%	0.009
Tumor size (>5cm VS <5cm)	2	0.14 (0.04-0.55)	Random	0.158; 50%	0.005
Tumor differentiation (well VS poor)	3	0.86 (0.36-2.09)	Random	0.01; 77%	0.75
Gender (male VS female)	3	1.14 (0.78-1.66)	Fixed	0.46; 0%	0.51

Three studies including 402 patients reported HRs for DFS. As shown in **Figure 2B** and **Table 3**. Due to the low heterogeneity (I^2^=0%), a fixed-effects model was used for the analysis. The combined data showed that high expression of ING3 indicated a better prognosis, with a pooled HR for DFS of 0.63 (95% CI: 0.37-0.88, *P* = 0). This implies that in individuals with malignancies, increased expression of ING3 may lead to improved clinical outcomes, while low expression of ING3 corresponds to a poor prognosis.

### Secondary endpoints: Relationship of ING3 to clinicopathological features

**Figure 3:**
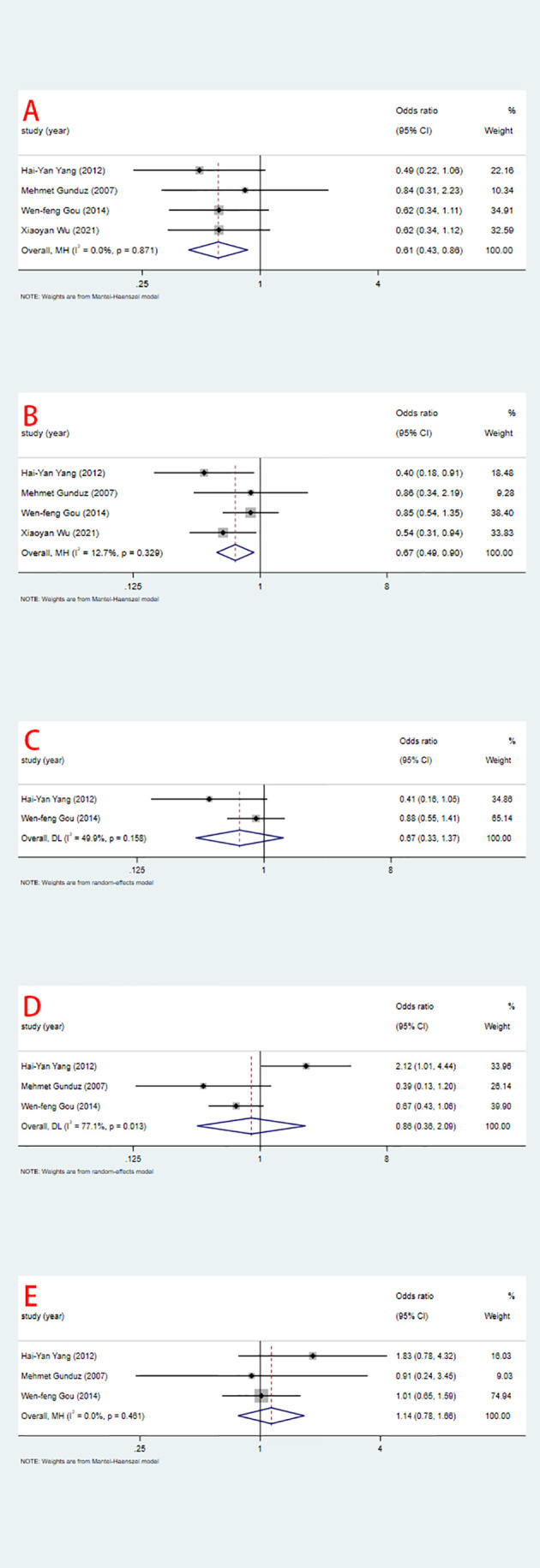
Relationship between ING3 expression and TNM stage **(A)**, lymph node metastasis **(B)**, tumor size **(C)**, tumor differentiation **(D)** and gender **(E)**.

In the seven included studies, the correlation between ING3 expression and clinicopathological features is shown in **Figure 3** and **Table 3**. We found that high expression of ING3 was negatively associated with TNM stage 3-4 (OR=0.61, 95% CI: 0.43-0.86) and lymph node metastasis (OR=0.67, 95% CI: 0.49-0.90). This means that in patients with low ING3 expression, tumors are more likely to progress to advanced stages or have lymph node metastases. Usually, this means a poor prognosis and leads to a poor clinical outcome. In the analysis of tumor size (OR=0.67, 95% CI: 0.33-1.37), tumor differentiation (OR=0.86, 95% CI: 0.36-2.09) and gender (OR=1.14, 95% CI: 0.78-1.66), they were all found to be unrelated to ING3 expression.

### Subgroup analysis

**Figure 4:**
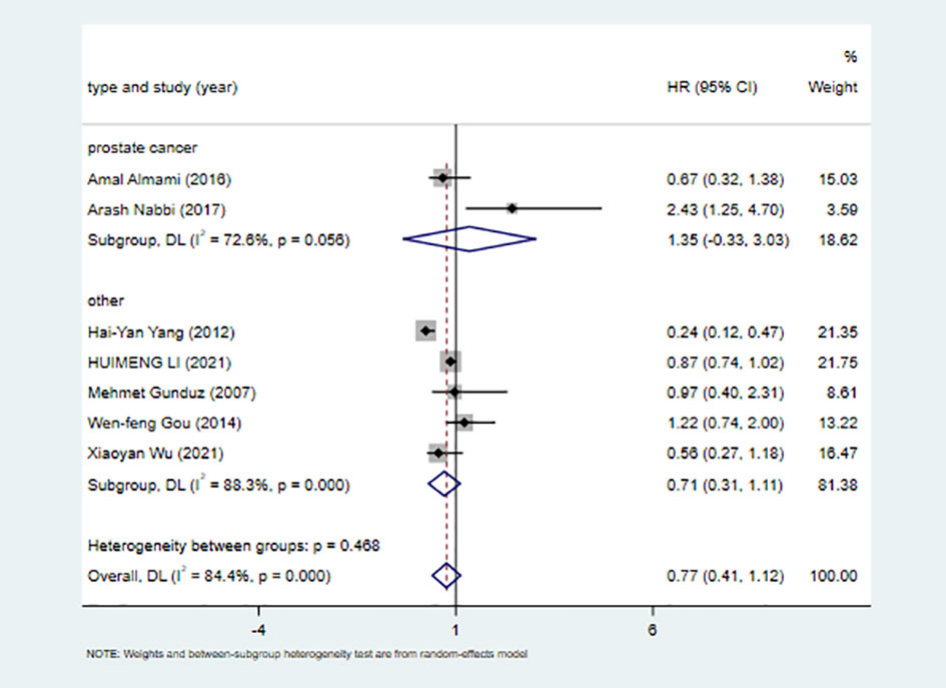
Subgroup analysis of tumor type.

To investigate whether ING3 plays a different role in prostate cancer, we performed a subgroup analysis by cancer type. The results of the subgroup analysis are shown in **Figure 4** and **Table 4**. We found a similar result in different types of cancer. There was significant heterogeneity in both subgroups, and overall survival was not associated with ING3 expression in either prostate cancer or other cancers.

**Table 4:** Subgroup analysis for the relationship between high ING3 expression and survival of cancer patients.

Subgroup	Studies	HR and 95% CI	Effects model	Heterogeneity (p; I^2^)	P value
Cancer types	7	0.77 (0.41-1.12)	Random	0; 84.4%	0
Prostate cancer	2	1.35 (-0.33-3.03)		0.056; 72.6%	0.115
Others	5	0.71 (0.31,1.11)		0; 88.3%	0.000

### Analysis of sensitivity

**Figure 5:**
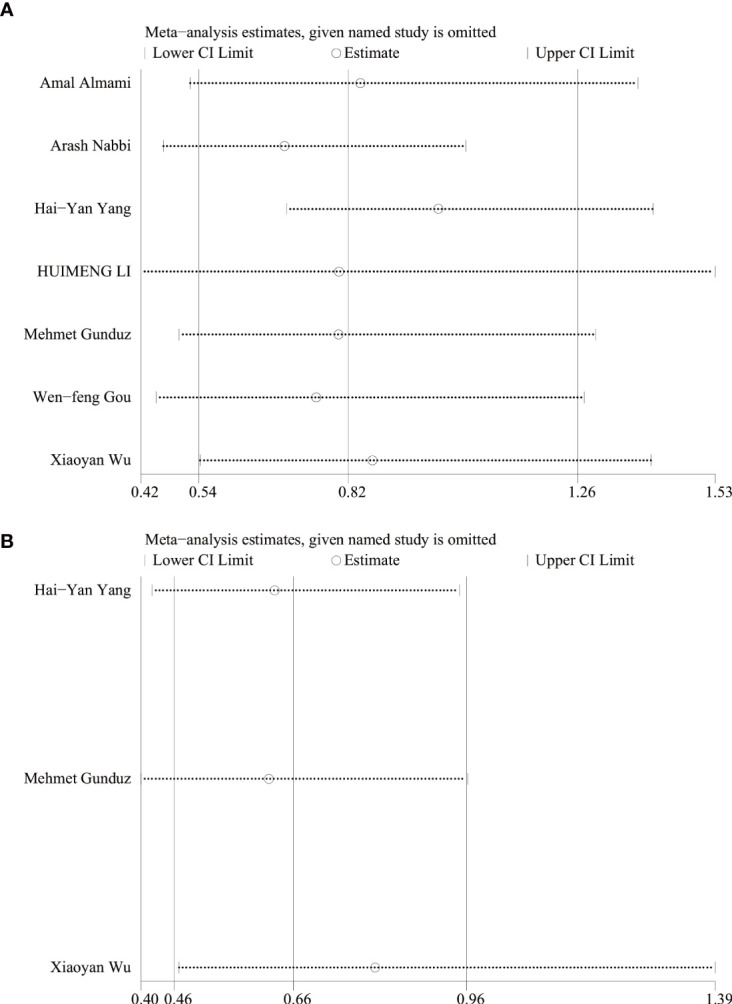
Sensitivity of systematic review and meta-analysis of OS **(A)** and DFS **(B)**.

**Figure 6:**
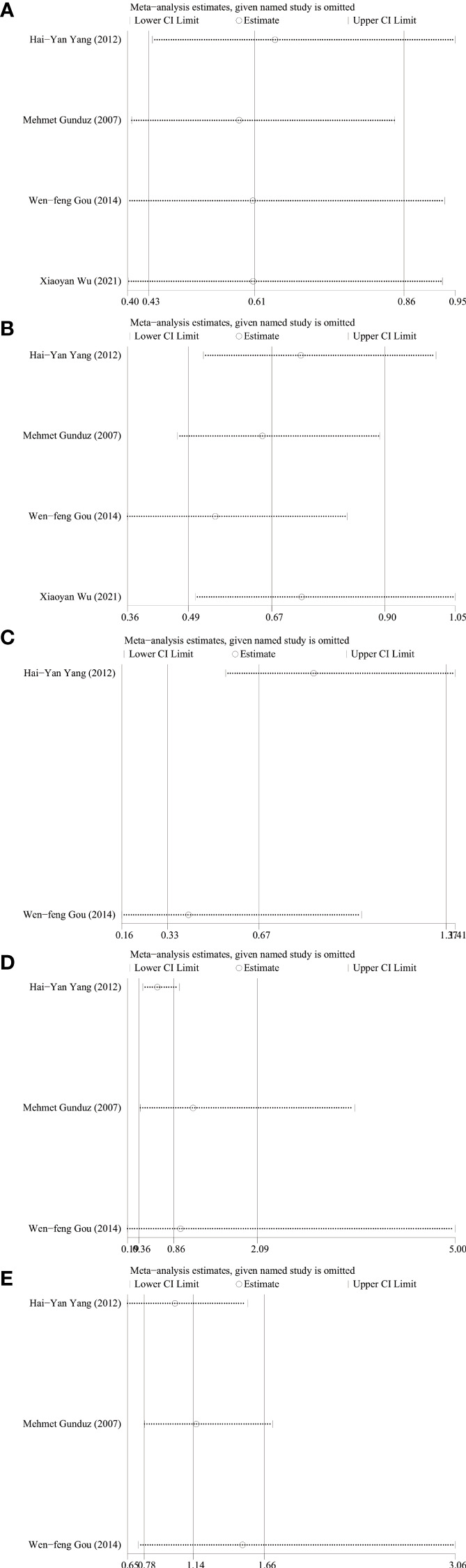
Sensitivity analysis for relationship between ING3 expression and TNM stage **(A)**, lymph node metastasis **(B)**, tumor size **(C)**, tumor differentiation **(D)** and gender **(E)**.

Sensitivity analysis was performed using STATA 17 software. The purpose of the sensitivity analysis was to determine whether any single study affected the overall results. We found that removing studies one by one did not significantly change the results, suggesting that the overall results were not influenced by individual studies. The HRs and 95% CIs varied in the range from 0.70 (95% CI: 0.47–1.05) to 0.99 (95% CI: 0.70–1.41) for overall survival (**Figure 5A**), and from 0.61 (95% CI: 0.40–0.96) to 0.80 (95% CI: 0.46–1.39) for disease-free survival (**Figure 5B**). The ORs and 95% CIs varied in the range from 0.58 (95% CI: 0.40–0.85) to 0.64 (95% CI: 0.44–0.95) for TNM stage (**Figure 6A**), from 0.54 (95% CI: 0.36–0.82) to 0.73 (95% CI: 0.51–1.05) for lymph node metastasis (**Figure 6B**), from 0.54 (95% CI: 0.36– 0.82) to 0.73 (95% CI: 0.51–1.05) for lymph node metastasis (**Figure 6B**), from 0.41 (95% CI: 0.16–1.05) to 0.88 (95% CI: 0.55–1.40) for tumor size (**Figure 6C**), from 0.62 (95% CI: 0.41– 0.95) to 1.15 (95% CI: 0.37–3.52) for tumor differentiation (**Figure 6D**), from 1.00 (95% CI: 0.65–1.54) to 1.50 (95% CI: 0.73–3.06) for gender (**Figure 6E**).

### Publication bias test

**Figure 7:**
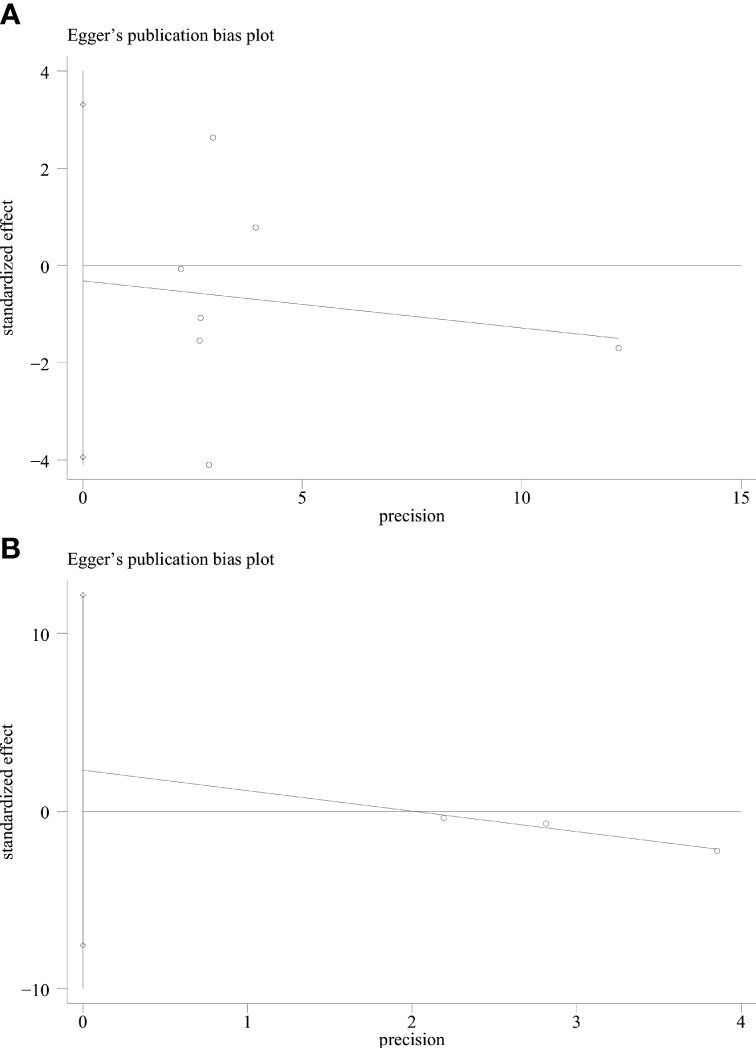
Egger’s test for OS **(A)** and DFS **(B)**.

**Figure 8:**
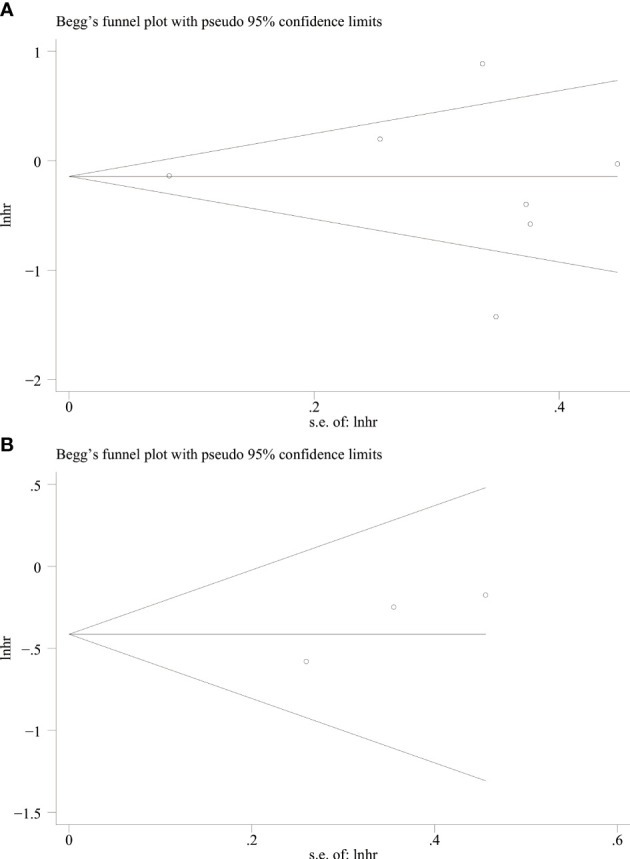
Begg’s funnel plot for OS **(A)** and DFS **(B)**.

**Figure 9:**
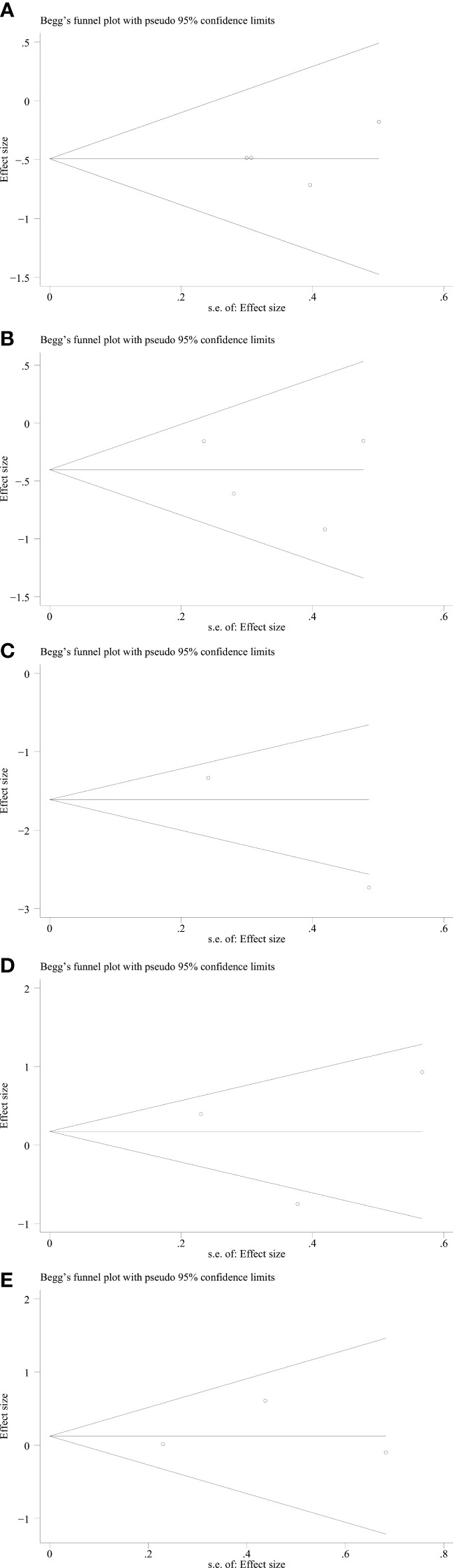
Begg’s funnel plot for relationship between ING3 expression and TNM stage **(A)**, lymph node metastasis **(B)**, tumor size **(C)**, tumor differentiation **(D)** and gender **(E)**.

We used Begg’s and Egger’s linear regression tests to detect any publication bias. The results of the Egger’s test are shown in (**Figure 7**). No asymmetry was observed in the funnel plot in the assessment of ING3 expression and OS (**Figure 8A**), DFS (**Figure 8B**), TNM stage (**Figure 9A**), lymph node metastasis (**Figure 9B**), tumor size (**Figure 9C**), tumor differentiation (**Figure 9D**), and gender (**Figure 9E**). No publication bias was observed in assessing ING3 expression and OS, DFS, TNM stage, lymph node metastasis, tumor size, tumor differentiation, and gender according to Begg’s test (*P* = 0.764, *P* = 0.296, *P* = 1.000, *P* = 0.734, *P* = 1.000, *P* = 1.000, and *P* = 1.000, respectively). A similar conclusion can be derived from Egger’s test (*P*=0.832, *P*=0.207, *P*=0.647, *P*=0.611, *P*=0.941, and *P*=0.773, respectively). **Table 5** shows the detailed data for Begg’s test and Egger’s test.

**Table 5:** Begg’s test and Egger’s test.

Clinicopathological characteristics	Begg’s test		Egger’s test		
	z	p	t	p	95% CL
Overall survival	0.30	0.764	-0.22	0.832	-3.940297, 3.311348
Disease-free survival	1.04	0.296	2.96	0.207	-7.55605, 12.15552
TNM stage (III-IV VS I-II)	-0.34	1.000	0.53	0.647	-5.388708, 6.916675
Lymph node metastasis (Yes VS No)	0.34	0.734	-0.60	0.611	-10.78117, 8.153391
Tumor size (>5cm VS <5cm)	0.00	1.000			
Tumor differentiation (well VS poor)	0.00	1.000	0.09	0.941	-62.21108, 63.13383
Gender (male VS female)	0.00	1.000	0.37	0.773	-20.30779, 21.53502

## Discussion

Cancer is becoming a growing public health problem worldwide. In 2021, the United States is anticipated to have 1,898,160 new cancer cases and 608,570 cancer deaths, making cancer the second leading cause of death in the country (33). Although the prognosis of cancer patients has improved substantially due to advances in medical therapy, the prognosis of the majority of patients remains dismal. One explanation is that most tumors have already progressed or metastasized by the time they are detected, which makes treatment extremely challenging. Another explanation is that oncologists may overestimate cancer patients’ prognoses, resulting in delayed or even missed conversations regarding patients’ goals and ultimately poor end-of-life care (34).

Cancer biomarkers can be classified into three types: predictive biomarkers, prognostic biomarkers and diagnostic biomarkers. Prognostic biomarkers can guide individualized treatment, assess efficacy, and optimize follow-up (35). Several prognostic biomarkers have been discovered, such as CA19-9 (36), MIR4435-2HG (37), AFP (38), and CEA (39). In recent years, with the development of technology, especially the advent of liquid biopsy (40), circulating tumor DNA (ctDNA) has become a new biomarker for the diagnosis of cancer (41, 42). However, the specificity and sensitivity of prognostic biomarkers remain low (43), and few biomarkers have entered clinical practice (35). Hence, there is an urgent need to discover prognostic biomarkers that might assist doctors in early cancer diagnosis and treatment (44, 45).

ING3 is the most unique member of the ING family. Unlike the high similarity exhibited between ING1, ING2, ING4 and ING5, ING3 has the least similarity to the others (46). Previous studies have reported that ING3 expression varies in different types of cancer. Almost all *in vitro* studies have shown that ING3 has the ability to inhibit growth (47), however, it was found that ING3 can function as an oncogene in prostate cancer (30). Nabbi et al. reported that ING3 may act as a coactivator of androgen receptor in prostate cancer (15). Since androgens can stimulate prostate cancer cell growth, this may lead to prostate cancer progression. Due to the controversial findings of existing studies, we conducted this meta-analysis and systematic review to investigate what role ING3 actually plays in cancer.

This systematic review and meta-analysis included seven studies involving 2,371 patients and five types of cancer. Our findings showed that high expression of ING3 was negatively associated with disease-free survival (HR=0.63, 95% CI: 0.37-0.88), a more advanced TNM stage (III-IV vs. I-II) (OR=0.61, 95% CI: 0.43-0.86) and lymph node metastasis (OR=0.67, 95% CI: 0.49-0.90). In other words, reduced expression of ING3 may lead to faster cancer progression, greater lymph node metastasis, and worse disease-free survival, all of which usually lead to poor prognosis. In the analysis of the relationship between ING3 expression and OS, we found no relationship between ING3 expression and OS, and a significant heterogeneity was observed. We assumed that the heterogeneity might come from the different types of cancer included in the study. We also found that ING3 expression did not correlate with clinicopathological data such as tumor size, tumor differentiation and gender. In the subgroup analysis, we found no significant difference between prostate cancer and other cancers. However, given the small number of included studies, this finding should be interpreted with caution. Sensitivity analysis showed that the pooled results were not affected by any single study, and after removing individual studies, the conclusions remained consistent with the main study findings. Begg’s test and Egger’s test showed no significant publication bias.

After considering all the results together, we conclude that ING3 expression predicts a better prognosis for cancer patients and can be used as a prognostic biomarker. ING3 may inhibit cancer progression through its PHD domains. PHD domains play a key role in the function of epigenetic regulatory proteins, which are known as chromatin remodeling factors and perform a function in the expression of genes (7). Malakhov et al. found that knockdown of the PHD domain resulted in epithelial-to-mesenchymal transition (EMT) and cellular senescence in LNCaP prostate cancer cell lines, suggesting that ING3 could limit the diffusion of cancer cells (48). ING3 may inhibit other types of cancer in the same way. In addition, the nucleolar translocation sequence (NTS) can aid in the translocation of ING proteins when DNA is damaged, and when it is mutated, it leads to a decrease in the level of apoptosis (49). Furthermore, ING3 expression was found to be associated with the expression levels of p300, p21 and acetylated p53. The interaction between ING3 and p300 can lead to the upregulation of acetylated p53 and induce cell cycle arrest and senescence associated with p53 (50). Although ING3 can act as a coactivator of the androgen receptor and thus contribute to the development of prostate cancer, it can still inhibit the progression of other types of cancer because they are rarely affected by androgens.

However, this study still has limitations. First, several HRs and 95% Cls were calculated using Kaplan-Meier survival curves, which may deviate from the actual situation. In addition, the number of included studies was small, and the number of studies was even smaller when it came to each type of cancer, so the results for some cancers lacked statistical significance. Finally, our study included different tumors and different treatments, due to the different pathogenesis of different cancers, which may lead to great heterogeneity.

Cancer biomarkers are key to the discovery and development of new cancer therapies. They are also an important factor in clinical practice as they can be used for risk assessment, diagnosis, prognosis and evaluation of efficacy. In addition, biomarkers are key to the success of precision medicine. The use of biomarkers that can identify and predict therapeutic response is essential in precision medicine (51). Overall, the findings of this study provide positive support for more research evaluating the expression levels of ING3 in cancer patients and may have practical clinical applications in treatment decision-making or early malignancy detection.

## Conclusion

This meta-analysis investigated the relationship between high expression of ING3 and the prognosis of various cancers. We found that high expression of ING3 was negatively associated with more advanced TNM stage, lymph node metastasis, and disease-free survival. This suggests that ING3 may be related to cancer prognosis and can be used as a prognostic biomarker. However, more studies are still needed to confirm these findings.

## Data availability statement

The original contributions presented in the study are included in the article/supplementary material. Further inquiries can be directed to the corresponding author.

## Author contributions

ZL developed the search strategy for the review and assisted with the method description. ZL, SX, and LC completed the article screening and completed the data extraction. ZL and LC created the PRISMA diagram. ZL and SX created the tables. ZL performed the meta-analysis. SH and XK helped interpret the results and correct grammatical errors in the article. All authors contributed to the article and approved the submitted version.
